# Thrombotic Thrombocytopenia after COVID-19 Vaccination: In Search of the Underlying Mechanism

**DOI:** 10.3390/vaccines9060559

**Published:** 2021-05-27

**Authors:** Piotr Rzymski, Bartłomiej Perek, Robert Flisiak

**Affiliations:** 1 Department of Environmental Medicine, Poznan University of Medical Sciences, 60-806 Poznań, Poland; 2 Integrated Science Association (ISA), Universal Scientific Education and Research Network (USERN), 60-806 Poznań, Poland; 3 Department of Cardiac Surgery and Transplantology, Poznan University of Medical Sciences, 61-848 Poznań, Poland; bperek@ump.edu.pl; 4 Department of Infectious Diseases and Hepatology, Medical University of Bialystok, 15-540 Białystok, Poland; robert.flisiak@umb.edu.pl

**Keywords:** vector vaccines, mRNA vaccines, thrombosis, thrombocytopenia, adverse effects, COVID-19

## Abstract

The rollout of COVID-19 vaccines brings hope for successful pandemic mitigation and getting the transmission of SARS-CoV-2 under control. The vaccines authorized in Europe displayed a good safety profile in the clinical trials. However, during their post-authorization use, unusual thrombotic events associated with thrombocytopenia have rarely been reported for vector vaccines. This led to the temporary suspension of the AZD1222 vaccine (Oxford/AstraZeneca) in various European countries and the Ad26.COV2 vaccine (Janssen/Johnson&Johnson) in the United States, with regulatory bodies launching investigations into potential causal associations. The thromboembolic reactions were also rarely reported after mRNA vaccines. The exact cause of these adverse effects remains to be elucidated. The present paper outlines the hypotheses on the mechanisms behind the very rare thrombotic thrombocytopenia reported after the COVID-19 vaccination, along with currently existing evidence and future research prospects. The following are discussed: (i) the role of antibodies against platelet factor 4 (PF4), (ii) the direct interaction between adenoviral vector and platelets, (iii) the cross-reactivity of antibodies against SARS-CoV-2 spike protein with PF4, (iv) cross-reactivity of anti-adenovirus antibodies and PF4, (v) interaction between spike protein and platelets, (vi) the platelet expression of spike protein and subsequent immune response, and (vii) the platelet expression of other adenoviral proteins and subsequent reactions. It is also plausible that thrombotic thrombocytopenia after the COVID-19 vaccine is multifactorial. The elucidation of the causes of these adverse events is pivotal in taking precautionary measures and managing vaccine hesitancy. It needs to be stressed, however, that the reported cases are currently sporadic and that the benefits of COVID-19 vaccines vastly outweigh their potential risks.

## 1. Introduction

The rollout of the COVID-19 vaccines brings hope that we can put an end to the pandemic. At the moment of writing, there are four vaccines authorized in the European Union: two developed using the mRNA platform (BNT162b by BioNTech/Pfizer, Germany, Mainz/New York, NY, USA and mRNA-1273 by Moderna, Cambridge, MA, USA) and two vector vaccines based on modified, replication-deficient (E1/E3-deletion) adenoviruses (AZD1222, ChAdOx1 nCov-19 by Oxford/AstraZeneca, UK/Sweden and Ad26.COV2.S by Janssen/Johnson&Johnson, Leiden, Netherlands/New Brunswick, NJ, USA). All of these vaccines were made available at previously unseen speed due to years of research and technological advances, the use of innovative platforms enabling rapid development of candidates, running multiple trials in parallel, significant funding, and help from regulatory institutions and their experts working at a higher pace [1,2,3]. The clinical trials have revealed a good safety profile with the reactogenicity represented mainly by short-term local (e.g., injection-site pain, redness, or swelling) and systemic (e.g., fatigue, headache, or fever) responses [4,5,6,7]. However, it should be stressed that despite thousands of participants, these studies, similarly to other clinical trials, were not designed to detect very rare side effects that may occur following the vaccine administration. Therefore, comprehensive safety monitoring and risk management through pharmacovigilance is pivotal following the vaccines’ authorization and needs to be continuously pursued during their general use in the population [8]. The cluster of specific side effects not reported during the clinical trials or their frequency exceeding the background levels requires investigation to understand whether the association with vaccination time is only of a correlative nature or whether a causal relationship is present.

The COVID-19 vaccination programs are conducted under extraordinary conditions and receive high traditional and online social media attention and coverage. Therefore, reports of severe events after the vaccination may rapidly cause public fear, vaccine hesitancy, political pressures, and inconsistent decisions [9,10]. Altogether, this leads to a need to elucidate as soon as possible whether a causal link between such events and vaccination exists, understand the mechanism of action, identify potential risk groups, and provide a mitigation strategy. In many instances, this may be a time-consuming and challenging task requiring different types of data derived from epidemiological observations, laboratory tests, autopsy analyses, and additionally conducted experiments.

The reports of thrombotic events occurring after the vaccination with AZD1222 have raised concerns over a possible causal relationship. At the beginning of April 2021, 169 cases of cerebral venous sinus thrombosis (CVST) and 53 cases of splanchnic vein thrombosis (SVT) were reported to EudraVigilance per 34 million individuals who received this vaccine in Europe, giving an overall reporting rate of 6.5 events per million [11]. This may still fall into the expected background rate levels, since the incidence of CVST and SVT has been reported in international studies at 2–16 per million per year and 1–7 per million per year, respectively [12,13,14,15]. One should, however, bear in mind that reporting rate cannot be directly compared to the incidence rate, while the reporting itself may vary across the countries and geographical regions depending on the efficiency of the national reporting systems. However, the thrombotic events reported after AZD1222 were represented mainly by CVST and SVT, while their peculiar feature included thrombocytopenia. This has led to suspending the AZD1222 rollout in several European countries [16]. The recent population-based cohort study in Denmark and Norway found an excess rate of venous thromboembolism, including CVST, in the group that received the first dose of AZD1222. However, as noted, the absolute risk of these events was small [17]. Meanwhile, six events of thrombotic thrombocytopenia, noted per 6.8 million doses administered, were also reported in the United States following the immunization with Ad26.COV2.S vaccine, leading to a temporary suspension of vaccine rollout [18]. Importantly, during the clinical trial program for Ad26.CoV2.S, there were 11 cases of venous thromboembolic events in the vaccinated group (compared to 3 cases in the placebo group) and a single case of CVST with thrombocytopenia in the vaccine recipient [19,20], while in the case of clinical trials of AZD1222, only four thrombotic events were noted [4]. As reported recently using data from US electronic health records, the incidence of CVST in 2 weeks following the mRNA vaccine (BNT162b2 or mRNA-1273) administration was 4.1 per million, which is relatively similar to a reporting rate of these events following AZD1222 vaccination in Europe. However, the incidence of portal vein thrombosis was 44.9 per million, 28-fold higher than according to the data collected on AZD1222 by the European Medicines Agency (EMA) [21,22]. Prior to this report, twenty cases of apparent secondary immune thrombocytopenia following the mRNA vaccination were reported and reviewed in the USA. The majority of these cases had no pre-existing thrombocytopenia. The investigation did not confirm nor exclude the possibility that these events were triggered by vaccine administration—it was noted that their incidence appears either less than or roughly comparable to the background level in the population [23]. The other study analyzed cases of arterial and venous thrombosis reported between 13 December 2020 and 16 March 2021 to Vigibase following the administration of COVID-19 vaccines [24]. The arterial thrombotic events were decidedly more common than venous thrombotic events for both mRNA vaccines. In the case of AZD1222, the proportion of venous and arterial thrombosis was evenly distributed, with five out of seven CVST cases accompanied by thrombocytopenia [24]. However, one should be aware of possible bias in reporting thrombosis and thrombocytopenia following the vaccination, as only clinically evident cases of such events are being submitted to the relevant databases. To understand the true prevalence, all vaccinated individuals would need to undergo routine monitoring of platelet counts and their indices (to detect thrombocytopenia) or imaging studies (to diagnose arterial and venous thrombosis), which is virtually impossible. Altogether, the rare cases of thrombotic thrombocytopenia associated in time with the COVID-19 vaccination require further and multidimensional studies.

Here, we elaborate on potential hypotheses on the mechanism behind these very rare cases of thrombotic thrombocytopenia following the COVID-19 vaccination and discuss the potential research avenues to explore. The following are discussed: (i) the role of antibodies against platelet factor 4 (PF4), (ii) the direct interaction between adenoviral vector and platelets, (iii) the cross-reactivity of anti-SARS-CoV-2 spike protein antibodies with PF4, (iv) the cross-reactivity of anti-adenovirus antibodies with PF4, (v) the interaction between spike protein and platelets, (vi) the platelet expression of spike protein and subsequent immune response, and (vii) the platelet expression of other adenoviral proteins and subsequent reactions. The elucidation of the causes of these adverse events is pivotal to taking precautionary measures and the managing of vaccine hesitancy related to particular or even all COVID-19 vaccines. However, it has to be stressed that the reported cases of thrombotic thrombocytopenia are currently sporadic, significantly less frequent than in COVID-19 [22], and even if they are resulting from the vaccination, the benefits of COVID-19 vaccines still vastly outweigh the potential risks.

## 2. Generation of Antibodies against Platelet Factor 4

Since the reported CVST and SVT events were often accompanied by thrombocytopenia, they resemble a clinical image seen during heparin-induced thrombocytopenia (HIT). The latter is an autoimmune complication following the administration of heparin that leads to the generation of pathogenic antibodies that bind the complex of heparin and platelet factor 4 (PF4), a cytokine released from the alpha-granules by the activated platelets. This complex, in turn, interacts with platelets’ FcγRIIA, subsequently shifting thrombocytes to a hypercoagulable state, causing the release of additional PF4 and promoting both arterial and venous thrombosis [25]. However, it is recognized that a similar mechanism can also occur following exposure to compounds other than heparin, such as polyvinyl phosphonate [26]. There are also reports of patients with a thromboembolic disorder resembling HIT with no history of heparin use and without identifiable causative factors [27,28]. The recently published case reports on thrombosis and thrombocytopenia were seen 5–24 days following administration of the AZD1222 vaccine, showing that these events closely resemble HIT. The patients presented with elevated d-dimer levels and low or normal fibrinogen levels and required hospitalization, while in some subjects the condition was fatal. As demonstrated, these patients, of whom some had no history of heparin use, tested positive for antibodies against PF4–heparin complex and on the platelet-activation assay in the presence of PF4 independent of heparin [29,30,31]. In one study, the antibodies were purified and also tested positively on a PF4 dependent platelet activation assay. Case reports of thrombotic thrombocytopenia 14 days after Ad26.COV2.S vaccination showed that despite a lack of antibodies against PF4-heparin complex, high levels of antibodies against PF4-polyanion were detected—one should cautiously note that the patient received heparin treatment prior to this testing [32]. All in all, the reported observations indicate that these thromboembolism events closely resemble HIT; therefore, the term vaccine-induced immune thrombotic thrombocytopenia (VITT) was coined to refer to them [29].

The reported events show a potential link with AZD1222 vaccination, although the possible mechanism behind them still requires further elucidation. As yet, it is unknown exactly what component of the vaccine is involved in the PF4 complex against which the antibodies are generated.

In HIT, the electrostatic interactions between positively charged PF4 and negatively charged heparin drive the formation of the PF4-heparin complex [33]. The surface of adenoviruses is negatively charged [34]; therefore, one could postulate that PF4 released from platelets can potentially form an immunogenic complex with adenoviral particles, further bound by IgG antibodies. Such a multimolecular complex could subsequently bind to platelets through the Fc receptor, activating thrombocytes and causing an additional release of PF4, leading to VITT.

Other authors have suggested that free adenoviral DNA in vector vaccines could trigger the VITT since nucleic acids have been shown in vivo to form complexes with PF4, which can induce antibodies against the PF4-heparin complex [35]. As yet, it is unknown whether free DNA is present in AZD1222 or Ad26.COV2.S vaccines. Moreover, such free DNA would be prone to degradation due to serum deoxyribonuclease (DNAse) activity [36]. However, it could be released from adenoviral particles following the innate immune system’s response to the viral vector [37,38]. It also requires further investigation to discover whether these rare cases of thrombotic thrombocytopenia can result from interactions with other ingredients present in adenoviral vaccines, such as ethylenediaminetetraacetic acid (EDTA). Although this compound is known to exert an anticoagulation effect [39], EDTA-dependent pseudothrombocytopenia has also rarely been reported [40,41,42]. As demonstrated, this phenomenon results from the generation of antiplatelet autoantibodies recognizing antigens on the platelet membrane modified by EDTA, which subsequently leads to platelet clumping [43,44]. However, one should note that EDTA is not an ingredient of any COVID-19 vaccine developed using the mRNA platform and is not used in Ad26.COV2.S vaccine. Therefore, its potential involvement in thrombotic events should only be taken into account in the case of AZD1222 vaccine.

It is unknown whether the investigated patients with VITT had pre-existing low levels of antibodies against PF4. It is important to note that many subjects who synthesize high levels of antibodies against the PF4-heparin complex do not develop HIT while being treated with heparin [45,46,47]. Therefore, the potential role of genetic factors in the observed rare cases of thrombotic thrombocytopenia after COVID-19 vaccination should be considered. It would be of interest to study PF4 polymorphism in patients with VITT, although it appears to be an unlikely contributor to HIT development [48]. In turn, genotype 131RR of the *FCGR2A* gene, encoding FcγRIIA, has been associated with an increased risk of thrombosis in subjects synthesizing significant levels of antibodies against the PF4-heparin complex [49,50]. Therefore, it is reasonable to investigate if patients homozygous for the FcγRIIA 131R allele are more susceptible to VITT. In addition, HIT risk has also been linked to the polymorphism of interleukin-10 promoter microsatellite as well as Pl^A2^ polymorphism of platelet glycoprotein IIIa [51,52]. Establishing whether these factors also play any role in the risk of VITT requires further studies.

## 3. Direct Interaction between Adenoviral Vector and Platelets

It is established that some adenoviruses can bind to the platelets using the coxsackie and adenovirus receptor (CAR), which represents an initial step for virus entry into thrombocytes [53]. The replication-deficient recombinant chimpanzee ChAdOx1 vector (the main component of the AZD1222 vaccine) has been shown to utilize CAR [54]. However, it has been noted that human adenovirus type 26 (the replication-deficient recombinant version of which is the main component of the Ad26.COV2.S vaccine) does not use CAR as a primary entry receptor. It has been suggested that CD46 plays such a role [55], although this has also recently been excluded [56]. Instead, sialic acid has been demonstrated to represent a primary cell receptor for human adenovirus type 26 [56]. Human platelets are known to differ in the content of sialic acid, which has been implicated in their aggregation and adhesion and may play a role in platelet disorders such as thrombocytopenia [57]. Moreover, the experimental in vitro studies have also shown that, in addition to the CAR pathway, ChAdOx1 has a fiber-dependent but CAR- and CD46-independent mechanism of cell attachment [54].

Therefore, the interactions of ChAdOx1 and Ad26.COV2.S vectors with platelets are plausible, especially since each intramuscularly injected dose of these vaccine contains 5 × 10^10^ viral particles [4,7]. Furthermore, as demonstrated using human adenovirus type 5 and human adenovirus type 3, the binding of adenoviral particles to circulating platelets can activate the latter and lead to their aggregation [58,59]. Activated platelets, in turn, release PF4 from the alpha-granules [60]. Therefore, at this moment, it cannot be ruled out that administration of an adenoviral vector vaccine can in some instances lead to the occurrence of adenoviral particles in the blood, binding of platelets, their activation, and subsequent release of PF4. However, the emergence of antiPF4 antibodies would first require PF4 to form a complex with the hitherto unknown vaccine component.

It is also of interest to understand whether the adenoviral-platelet complex can trigger the autoimmune response by itself, particularly in the presence of the pre-existing antibodies against PF4. Notably, previous studies in rodents, rabbits, and non-human primates have shown that intravenous administration of adenoviral vectors can lead to acute thrombocytopenia and coagulopathy with onset within 24 h [61,62,63]. As also demonstrated, these vectors can activate platelets and induce platelet-leukocyte aggregation formation, with the von Willebrand factor (WF) playing a critical role in the initiation of these processes [61]. However, a case of pulmonary embolism and thrombocytopenia following AZD1222 administration reported no changes to plasma WF [64], while other studies did not investigate it [29,30,31].

As already mentioned, adenoviruses can utilize CAR and other receptors to infect platelets. In vivo studies have shown that the presence of high adenoviral load in the blood can lead to acute thrombocytopenia [61,62,63]. Intravenous administration of adenovirus type 5 in hCD46Ge transgenic mice resulted in platelet activation degranulation, cytokine release, binding of platelets to endothelial cells, and subsequent activation of the latter, eventually promoting the microthrombus formation [59]. Moreover, in vitro research employing E1/E3-deleted, replication-deficient human adenovirus type 5 can activate human platelets, induce rapid expression of P-selectin, and potentate ADP-induced platelet aggregation [65]. So far, there are no similar studies on ChAdOx1 adenovirus and human adenovirus type 26, utilized in AZD1222 and Ad26.COV2.S vaccines, respectively, although given the circumstances, they are urgently needed. Both of these vaccines are administrated intramuscularly, and according to the biodistribution study in BALB/c mice, the highest number of viral copies is noted in skeletal muscle at the site of administration, with low levels sporadically found in other tissues—heart, liver, lymph node, ovary, and testes (study 0841MV38.001) [66]. Rare translocation of adenoviral vector outside the injection site may potentially result in its interaction with platelets—a hypothesis worth further exploration.

## 4. Cross-Reactivity of Anti-SARS-Cov-2 Spike Protein Antibodies with Platelet Factor 4

Cross-reactivity occurs when an antibody directed against one specific antigen successfully recognizes epitopes of other antigens [67]. For example, anti-nucleocapsid antibodies generated after SARS-CoV-1 infection can cross-react with interleukin-11, an anti-inflammatory cytokine derived from bone marrow stromal cells [68]. Potential cross-reactivity of anti-SARS-CoV-2 spike antibodies and different tissue proteins has also been postulated and encompassed: transglutaminase 3, transglutaminase 2, myelin basic protein, mitochondria, nuclear antigen, α-myosin, thyroid peroxidase, collagen, claudin 5+6, and S100B [69]. This leads to the question of whether the antibodies against spike protein could cross-react with PF4. However, such a mechanism is questionable; otherwise, prior to vaccine rollout, the increase in thrombotic thrombocytopenia should be reported in convalescent patients. It is true that thromboembolic events emerging from COVID-19 are behind the sudden deterioration and death, although it has been proposed that they arise from the direct platelet stimulation by SARS-CoV-2 and spike protein binding angiotensin-converting enzyme 2 (ACE2) receptor, as well as endothelial damage due to cytokine storms [70,71].

The potential cross-reactivity of the anti-SARS-CoV-2 spike protein antibodies with PF4 has recently been investigated [72]. As shown using the prediction tools and 3D-modelling, three motifs of the spike protein sequences shared a possible immunogenic epitope with PF4, although the experiments with purified recombinant spike protein, purified PF4, and affinity-purified anti-PF4 antibodies collected from serum samples of individuals vaccinated with AZD1222 who developed thrombotic thrombocytopenia found no evidence for cross-reactivity [72]. This report is reassuring that SARS-CoV-2 spike protein remains an optimal immunological target for vaccines.

One should, however, note the distinctive difference in spike protein encoded by both mRNA vaccines (BNT162b2 and mRNA-1273), Ad26.COV2 vector vaccine, and AZD1222. While in all cases, the sequence encoding the prefusion conformation of spike protein is delivered, the former three utilize the stabilized “up” state of the receptor-binding domain [73,74,75]. Contrary to this, AZD1222 uses ChAdOx1 adenovirus vector encoding unstabilized prefusion conformation and, as shown experimentally in U2OS cells, the majority of spike protein on their surface was presented in a prefusion conformation. However, low levels of post-fusion spike protein were also shown [76]. Whether this may play any role in humoral responses and potential cross-reactivity remains to be tested.

## 5. Cross-Reactivity of Anti-Adenovirus Antibodies with Platelet Factor 4

It is known that pre-existing anti-vector antibodies introduce a significant limitation to the use of vector vaccines, particularly when utilizing recombinant human viruses. For example, the percentage of individuals in the African, American and European population displaying neutralizing antibodies against human adenovirus type 5 amounts to >65%, >37% and >6%, respectively [77,78,79,80]. This can be partially overcome by using less immunogenic adenoviral vectors such as human adenovirus type 26 or employing animal-derived adenoviruses [81]. However, such a strategy does not limit the possibility of the generation of anti-vector antibodies post-vaccination. As shown for AZD1222, anti-ChAdOx1 antibodies increased over the 28 days following first dose administration, and remained relatively stable for at least 84 days [82]. Whether these antibodies play a role in the observed VITT requires detailed studies. This may be achieved experimentally via isolation and purification of anti-vector antibodies and their incubation with human platelets and the use of tools to predict whether PF4 epitopes (or epitopes of other surface platelet factors) can be recognized and bound by these antibodies.

## 6. Interaction between Spike Protein and Platelets

Severe COVID-19 is often associated with a hypercoagulable state and thrombosis. The patients present reduced primary production of platelets, elevated destruction of circulating thrombocytes leading to thrombocytopenia, as well as endotheliopathy activating the complement system and contributing to the release of pro-inflammatory cytokines. All this can lead to blood coagulation in various organs such as the lung, liver, heart, brain, and kidney [83,84]. Notably, it has been evidenced that, similar to many other viruses (e.g., influenza virus), SARS-CoV-2 can directly activate platelets and subsequently trigger uncontrolled coagulation cascades and tissue injury. This is because they robustly express ACE2, a host receptor, and transmembrane protease serine 2 (TMPRSS2) that proteolytically cleaves spike protein at the S2′ site [70,85]. SARS-CoV-2 has been shown to potentiate platelet aggregation in response to ADP, collagen, and thrombin, and even more importantly, a similar effect was noted in the presence of purified spike protein. Moreover, spike protein was found to induce integrin αIIbβ3 activation and P-selectin expression in the absence of agonists [70]. This clearly shows that interactions between spike protein and ACE2 on the platelet surface are sufficient to potentate the prothrombotic function. Therefore, this finding leads to the question of whether spike protein produced following vaccination with vector or mRNA vaccines can (and how frequently) be secreted in free form, occur in the blood, and possibly interact with platelets in a similar manner. Following the vaccination with vector and mRNA vaccines, and production of spike protein, it is further degraded to antigenic peptide and presented to cytotoxic T lymphocytes through the major histocompatibility complex (MHC) I pathway, but they can also be released and taken by dendritic cells and further presented to helper T cells and B cells through the MHC II pathway [86]. It cannot, therefore, be ruled out that sporadic escape of spike protein and its transport can occur. To understand whether this is truly the case, and whether this could lead to interaction with platelets, investigations determining the spike protein content in blood samples are required.

## 7. Platelet Expression of Spike Protein

In addition, it cannot be excluded at this moment that sporadic infection of platelets with an adenoviral vector can occur. It is plausible since the platelets express CAR and salic acid that can be utilized by the adenoviruses for cell entry. As already mentioned, the translocation of ChAdOx1 adenoviral vector outside the administration site as far as the heart, liver, lymph node, ovary, and testes has rarely been noted (study 0841MV38.001) [66]. However, platelets infected with adenoviral vector will not express spike protein since they exist in an anucleate state and are DNA transcription incompetent [87]. Instead, its expression would be possible in megakaryocytes, platelet precursors located in the bone marrow, in which transcriptional production of RNA can occur [88]. Such possibility can be investigated in vitro since the mature megakaryocytes can be isolated, proliferated, and differentiated from human peripheral blood mononuclear cells [89]. Whether their infection with adenoviral vector and expression of spike protein can lead to immune reactions and thrombocytopenia would require further in vivo studies. The mRNA vaccines can also potentially be transported to platelets via endocytosis. An in vivo biodistribution study of BNT162b2 has revealed that spike protein can be expressed at low levels and transiently in the liver (study R-20-0072) [90]. It therefore remains to be studied whether the platelets (and megakaryocytes) can serve as target cells of COVID-19 vector and/or mRNA vaccines, leading to spike protein synthesis and its presentation in the context of MHC I [91] and the triggering of a cytotoxic response by CD^8+^ cells.

## 8. Platelet Expression of Adenoviral Proteins

In case of sporadic infection of megakaryocytes with adenoviral vector, it remains to be elucidated whether, in addition to gene encoding SARS-CoV-2 spike protein, the expression of genes encoding adenoviral proteins can occur [92]. As already shown using in vitro experiments, replication-deficient adenoviral vectors lacking E1 and E3 regions, e.g., ChAdOx1, can still express a wide array of structural and non-structural proteins, in addition to a heterologous gene product. However, this phenomenon appears to be restricted only to selected cell types as it was observed at a low level in A549 cells (a human lung epithelial-like continuous-line derived from carcinomatous tissue) but was nearly absent in MRC-5 cells (human lung fibroblast-like line) [92]. Neither is it clear whether the expression of different adenoviral proteins also occurs after vaccine administration nor in what cell types it can take place. However, it appears reasonable to study such a possibility in various human cells, e.g., megakaryocytes, and, if confirmed, understand the potential consequences, especially in the context of thrombotic thrombocytopenia risk.

## 9. Conclusions

This paper offers selected hypotheses behind the potential mechanism linking rare blood clotting events observed after administering the vector and mRNA vaccines against COVID-19. Based on the provided discussion, the evidence is building toward the role of pathologic antibodies to PF4. However, it remains to elucidate which component can form a complex with PF4 (spike protein appears unlikely due to no affinity) and whether the genetic predispositions (e.g., FcγRIIA polymorphism) may play a role in immune response leading to thrombotic thrombocytopenia after vaccination. At the same time, further research should, in particular, test the potential interaction between adenoviral vector and human platelets, take into account that other components of the vaccine may be involved, explore the possibility of cross-reactivity between anti-vector antibodies and PF4, and elucidate whether spike protein interaction with platelets can lead to an antiplatelet immune reaction. One should bear in mind the possibility that the causative factor may not be homogeneous in all cases of so-called VITT and that not all of these cases may have a causal relationship with vaccination. Although elucidating the causative mechanism after vaccination is challenging and time-consuming, the unprecedented research efforts in response to the COVID-19 pandemic raise a hope that causes of the observed rare cases of thrombotic thrombocytopenia after COVID-19 vaccination will eventually be well understood.

## References

[1] Burgos R.M., Badowski M.E., Drwiega E., Ghassemi S., Griffith N., Herald F., Johnson M., Smith R.O., Michienzi S.M. (2021). The race to a COVID-19 vaccine: Opportunities and challenges in development and distribution. Drugs Context.

[2] Defendi H.G.T., da Silva Madeira L., Borschiver S. (2021). Analysis of the COVID-19 Vaccine Development Process: An Exploratory Study of Accelerating Factors and Innovative Environments. J. Pharm. Innov..

[3] Nowakowska J., Sobocińska J., Lewicki M., Lemańska Ż., Rzymski P. (2020). When science goes viral: The research response during three months of the COVID-19 outbreak. Biomed. Pharmacother..

[4] Voysey M., Clemens S.A.C., Madhi S.A., Weckx L.Y., Folegatti P.M., Aley P.K., Angus B., Baillie V.L., Barnabas S.L., Bhorat Q.E. (2021). Safety and efficacy of the ChAdOx1 nCoV-19 vaccine (AZD1222) against SARS-CoV-2: An interim analysis of four randomised controlled trials in Brazil, South Africa, and the UK. Lancet.

[5] Polack F.P., Thomas S.J., Kitchin N., Absalon J., Gurtman A., Lockhart S., Perez J.L., Pérez Marc G., Moreira E.D., Zerbini C. (2020). Safety and Efficacy of the BNT162b2 mRNA Covid-19 Vaccine. N. Engl. J. Med..

[6] Baden L.R., El Sahly H.M., Essink B., Kotloff K., Frey S., Novak R., Diemert D., Spector S.A., Rouphael N., Creech C.B. (2020). Efficacy and Safety of the mRNA-1273 SARS-CoV-2 Vaccine. N. Engl. J. Med..

[7] Sadoff J., Le Gars M., Shukarev G., Heerwegh D., Truyers C., de Groot A.M., Stoop J., Tete S., Van Damme W., Leroux-Roels I. (2021). Interim Results of a Phase 1-2a Trial of Ad26.COV2.S Covid-19 Vaccine. N. Engl. J. Med..

[8] Excler J.-L., Saville M., Berkley S., Kim J.H. (2021). Vaccine development for emerging infectious diseases. Nat. Med..

[9] Rzymski P., Borkowski L., Drąg M., Flisiak R., Jemielity J., Krajewski J., Mastalerz-Migas A., Matyja A., Pyrć K., Simon K. (2021). The Strategies to Support the COVID-19 Vaccination with Evidence-Based Communication and Tackling Misinformation. Vaccines.

[10] Rzymski P., Zeyland J., Poniedziałek B., Małecka I., Wysocki J. (2021). The Perception and Attitudes toward COVID-19 Vaccines: A Cross-Sectional Study in Poland. Vaccines.

[11] European Medicines Agency. AstraZeneca’s COVID-19 Vaccine: EMA Finds Possible Link to Very Rare Cases of Unusual Blood Clots with Low Blood Platelets. https://www.ema.europa.eu/en/news/astrazenecas-covid-19-vaccine-ema-finds-possible-link-very-rare-cases-unusual-blood-clots-low-blood.

[12] Capecchi M., Abbattista M., Martinelli I. (2018). Cerebral venous sinus thrombosis. J. Thromb. Haemost..

[13] Coutinho J.M., Zuurbier S.M., Aramideh M., Stam J. (2012). The incidence of cerebral venous thrombosis: A cross-sectional study. Stroke.

[14] Devasagayam S., Wyatt B., Leyden J., Kleinig T. (2016). Cerebral Venous Sinus Thrombosis Incidence Is Higher Than Previously Thought: A Retrospective Population-Based Study. Stroke.

[15] Valeriani E., Riva N., Di Nisio M., Ageno W. (2019). Splanchnic Vein Thrombosis: Current Perspectives. Vasc. Health Risk Manag..

[16] Wise J. (2021). Covid-19: European countries suspend use of Oxford-AstraZeneca vaccine after reports of blood clots. BMJ.

[17] Pottegård A., Lund L.C., Karlstad Ø., Dahl J., Andersen M., Hallas J., Lidegaard Ø., Tapia G., Gulseth H.L., Ruiz P.L.-D. (2021). Arterial events, venous thromboembolism, thrombocytopenia, and bleeding after vaccination with Oxford-AstraZeneca ChAdOx1-S in Denmark and Norway: Population based cohort study. BMJ.

[18] Mahase E. (2021). Covid-19: US suspends Johnson and Johnson vaccine rollout over blood clots. BMJ.

[19] Sadoff J., Gray G., Vandebosch A., Cárdenas V., Shukarev G., Grinsztejn B., Goepfert P.A., Truyers C., Fennema H., Spiessens B. (2021). Safety and Efficacy of Single-Dose Ad26.COV2.S Vaccine against Covid-19. N. Engl. J. Med..

[20] Sadoff J., Davis K., Douoguih M. (2021). Thrombotic Thrombocytopenia after Ad26.COV2.S Vaccination—Response from the Manufacturer. N. Engl. J. Med..

[21] Taquet M., Husain M., Geddes J.R., Luciano S., Harrison P.J. Cerebral Venous Thrombosis: A Retrospective Cohort Study of 513 284 Confirmed COVID-19 Cases and a Comparison with 489 871 People Receiving a COVID-19 mRNA Vaccine. Version 2. https://osf.io/a9jdq/.

[22] Torjesen I. (2021). Covid-19: Risk of cerebral blood clots from disease is 10 times that from vaccination, study finds. BMJ.

[23] Lee E.-J., Cines D.B., Gernsheimer T., Kessler C., Michel M., Tarantino M.D., Semple J.W., Arnold D.M., Godeau B., Lambert M.P. (2021). Thrombocytopenia following Pfizer and Moderna SARS-CoV-2 vaccination. Am. J. Hematol..

[24] Smadja D.M., Yue Q.-Y., Chocron R., Sanchez O., Lillo-Le Louet A. (2021). Vaccination against COVID-19: Insight from arterial and venous thrombosis occurrence using data from VigiBase. Eur. Respir. J..

[25] Arepally G.M. (2017). Heparin-induced thrombocytopenia. Blood.

[26] Visentin G.P., Moghaddam M., Beery S.E., McFarland J.G., Aster R.H. (2001). Heparin is not required for detection of antibodies associated with heparin-induced thrombocytopenia/thrombosis. J. Lab. Clin. Med..

[27] Okata T., Miyata S., Miyashita F., Maeda T., Toyoda K. (2015). Spontaneous heparin-induced thrombocytopenia syndrome without any proximate heparin exposure, infection, or inflammatory condition: Atypical clinical features with heparin-dependent platelet activating antibodies. Platelets.

[28] Miyata S. (2016). Spontaneous heparin-induced thrombocytopenia (HIT) syndrome: HIT without any heparin exposure. [Rinsho Ketsueki] Jpn. J. Clin. Hematol..

[29] Schultz N.H., Sørvoll I.H., Michelsen A.E., Munthe L.A., Lund-Johansen F., Ahlen M.T., Wiedmann M., Aamodt A.H., Skattør T.H., Tjønnfjord G.E. (2021). Thrombosis and Thrombocytopenia after ChAdOx1 nCoV-19 Vaccination. N. Engl. J. Med..

[30] Greinacher A., Thiele T., Warkentin T.E., Weisser K., Kyrle P.A., Eichinger S. (2021). Thrombotic Thrombocytopenia after ChAdOx1 nCov-19 Vaccination. N. Engl. J. Med..

[31] Scully M., Singh D., Lown R., Poles A., Solomon T., Levi M., Goldblatt D., Kotoucek P., Thomas W., Lester W. (2021). Pathologic Antibodies to Platelet Factor 4 after ChAdOx1 nCoV-19 Vaccination. N. Engl. J. Med..

[32] Muir K.-L., Kallam A., Koepsell S.A., Gundabolu K. (2021). Thrombotic Thrombocytopenia after Ad26.COV2.S Vaccination. N. Engl. J. Med..

[33] Suvarna S., Espinasse B., Qi R., Lubica R., Poncz M., Cines D.B., Wiesner M.R., Arepally G.M. (2007). Determinants of PF4/heparin immunogenicity. Blood.

[34] Kim S.-Y., Kwon W.-A., Shin S.-P., Seo H.K., Lim S.-J., Jung Y.-S., Han H.-K., Jeong K.-C., Lee S.-J. (2018). Electrostatic interaction of tumor-targeting adenoviruses with aminoclay acquires enhanced infectivity to tumor cells inside the bladder and has better cytotoxic activity. Drug Deliv..

[35] Jaax M.E., Krauel K., Marschall T., Brandt S., Gansler J., Fürll B., Appel B., Fischer S., Block S., Helm C.A. (2013). Complex formation with nucleic acids and aptamers alters the antigenic properties of platelet factor 4. Blood.

[36] Tinazzi E., Puccetti A., Gerli R., Rigo A., Migliorini P., Simeoni S., Beri R., Dolcino M., Martinelli N., Corrocher R. (2009). Serum DNase I, soluble Fas/FasL levels and cell surface Fas expression in patients with SLE: A possible explanation for the lack of efficacy of hrDNase I treatment. Int. Immunol..

[37] Atasheva S., Shayakhmetov D.M., Curiel D.T. (2016). 14-Innate Immune Response to Adenovirus Vector Administration In Vivo. Adenoviral Vectors for Gene Therapy.

[38] Muruve D.A. (2004). The innate immune response to adenovirus vectors. Hum. Gene Ther..

[39] Banfi G., Salvagno G.L., Lippi G. (2007). The role of ethylenediamine tetraacetic acid (EDTA) as in vitro anticoagulant for diagnostic purposes. Clin. Chem. Lab. Med..

[40] Akbayram S., Dogan M., Akgun C., Caksen H., Oner A.F. (2011). EDTA-dependent pseudothrombocytopenia in a child. Clin. Appl. Thromb./Hemost..

[41] Ahn H.L., Jo Y.I., Choi Y.S., Lee J.Y., Lee H.W., Kim S.R., Sim J., Lee W., Jin C.J. (2002). EDTA-dependent pseudothrombocytopenia confirmed by supplementation of kanamycin; a case report. Korean J. Intern. Med..

[42] Berkman N., Michaeli Y., Or R., Eldor A. (1991). EDTA-dependent pseudothrombocytopenia: A clinical study of 18 patients and a review of the literature. Am. J. Hematol..

[43] Bizzaro N. (1995). EDTA-dependent pseudothrombocytopenia: A clinical and epidemiological study of 112 cases, with 10-year follow-up. Am. J. Hematol..

[44] Pegels J.G., Bruynes E.C., Engelfriet C.P., von dem Borne A.E. (1982). Pseudothrombocytopenia: An immunologic study on platelet antibodies dependent on ethylene diamine tetra-acetate. Blood.

[45] Warkentin T.E., Levine M.N., Hirsh J., Horsewood P., Roberts R.S., Gent M., Kelton J.G. (1995). Heparin-induced thrombocytopenia in patients treated with low-molecular-weight heparin or unfractionated heparin. N. Engl. J. Med..

[46] Warkentin T.E., Cook R.J., Marder V.J., Sheppard J.A., Moore J.C., Eriksson B.I., Greinacher A., Kelton J.G. (2005). Anti-platelet factor 4/heparin antibodies in orthopedic surgery patients receiving antithrombotic prophylaxis with fondaparinux or enoxaparin. Blood.

[47] Pouplard C., May M.A., Iochmann S., Amiral J., Vissac A.M., Marchand M., Gruel Y. (1999). Antibodies to platelet factor 4-heparin after cardiopulmonary bypass in patients anticoagulated with unfractionated heparin or a low-molecular-weight heparin: Clinical implications for heparin-induced thrombocytopenia. Circulation.

[48] Horsewood P., Kelton J.G. (2000). Investigation of a platelet factor 4 polymorphism on the immune response in patients with heparin-induced thrombocytopenia. Platelets.

[49] Arepally G., McKenzie S.E., Jiang X.-M., Poncz M., Cines D.B. (1997). FcγRIIA H/R131 Polymorphism, Subclass-Specific IgG Anti-Heparin/Platelet Factor 4 Antibodies and Clinical Course in Patients with Heparin-Induced Thrombocytopenia and Thrombosis. Blood.

[50] Rollin J., Pouplard C., Cheng Sung H., Leroux D., Saada A., Gouilleux-Gruart V., Thibault G., Gruel Y. (2015). Increased risk of thrombosis in FcγRIIA 131RR patients with HIT due to defective control of platelet activation by plasma IgG2. Blood.

[51] Pouplard C., Cornillet-Lefebvre P., Attaoua R., Leroux D., Lecocq-Lafon C., Rollin J., Grigorescu F., Nguyen P., Gruel Y. (2012). Interleukin-10 promoter microsatellite polymorphisms influence the immune response to heparin and the risk of heparin-induced thrombocytopenia. Thromb. Res..

[52] Harris K., Nguyen P., Van Cott E.M. (2008). Platelet PlA2 Polymorphism and the risk for thrombosis in heparin-induced thrombocytopenia. Am. J. Clin. Pathol..

[53] Gupalo E., Buriachkovskaia L., Othman M. (2011). Human platelets express CAR with localization at the sites of intercellular interaction. Virol. J..

[54] Dicks M.D.J., Spencer A.J., Coughlan L., Bauza K., Gilbert S.C., Hill A.V.S., Cottingham M.G. (2015). Differential immunogenicity between HAdV-5 and chimpanzee adenovirus vector ChAdOx1 is independent of fiber and penton RGD loop sequences in mice. Sci. Rep..

[55] Li H., Rhee E.G., Masek-Hammerman K., Teigler J.E., Abbink P., Barouch D.H. (2012). Adenovirus serotype 26 utilizes CD46 as a primary cellular receptor and only transiently activates T lymphocytes following vaccination of rhesus monkeys. J. Virol..

[56] Baker A.T., Mundy R.M., Davies J.A., Rizkallah P.J., Parker A.L. (2019). Human adenovirus type 26 uses sialic acid–bearing glycans as a primary cell entry receptor. Sci. Adv..

[57] Crook M. (1991). Sialic Acid: Its importance to platelet function in health and disease. Platelets.

[58] Jin Y.-Y., Yu X.-N., Qu Z.-Y., Zhang A.-A., Xing Y.L., Jiang L.-X., Shang L., Wang Y.-C. (2014). Adenovirus type 3 induces platelet activation in vitro. Mol. Med. Rep..

[59] Stone D., Liu Y., Shayakhmetov D., Li Z.-Y., Ni S., Lieber A. (2007). Adenovirus-Platelet Interaction in Blood Causes Virus Sequestration to the Reticuloendothelial System of the Liver. J. Virol..

[60] Arepally G., Poncz M., Cines D.B., Shoenfeld Y., Gershwin M.E., Meroni P.L. (2007). Autoantibodies in heparin-induced thrombocytopenia. Autoantibodies.

[61] Othman M., Labelle A., Mazzetti I., Elbatarny H.S., Lillicrap D. (2007). Adenovirus-induced thrombocytopenia: The role of von Willebrand factor and P-selectin in mediating accelerated platelet clearance. Blood.

[62] Lozier J.N., Csako G., Mondoro T.H., Krizek D.M., Metzger M.E., Costello R., Vostal J.G., Rick M.E., Donahue R.E., Morgan R.A. (2002). Toxicity of a first-generation adenoviral vector in rhesus macaques. Hum. Gene Ther..

[63] Cichon G., Schmidt H.H., Benhidjeb T., Löser P., Ziemer S., Haas R., Grewe N., Schnieders F., Heeren J., Manns M.P. (1999). Intravenous administration of recombinant adenoviruses causes thrombocytopenia, anemia and erythroblastosis in rabbits. J. Gene Med..

[64] Muster V., Gary T., Raggam R.B., WÖlfler A., Brodmann M. (2021). Pulmonary embolism and thrombocytopenia following ChAdOx1 vaccination. Lancet.

[65] Gupalo E., Kuk C., Qadura M., Buriachkovskaia L., Othman M. (2013). Platelet-adenovirus vs. inert particles interaction: Effect on aggregation and the role of platelet membrane receptors. Platelets.

[66] Medicines & Healthcare Products Regulatory Agency. Summary of the Public Assessment Report for AstraZeneca COVID-19 Vaccine. https://www.gov.uk/government/publications/regulatory-approval-of-covid-19-vaccine-astrazeneca/summary-of-the-public-assessment-report-for-astrazeneca-covid-19-vaccine.

[67] Sadrzadeh H., Baskin L., Kline G., Sadrzadeh H., Kline G. (2017). Variables affecting endocrine tests results, errors prevention and mitigation. Endocrine Biomarkers.

[68] Cheng M., Chan C.W.L., Cheung R.C.F., Bikkavilli R.K., Zhao Q., Au S.W.N., Chan P.K.S., Lee S.S.T., Cheng G., Ho W.K.K. (2005). Cross-reactivity of antibody against SARS-coronavirus nucleocapsid protein with IL-11. Biochem. Biophys. Res. Commun..

[69] Vojdani A., Kharrazian D. (2020). Potential antigenic cross-reactivity between SARS-CoV-2 and human tissue with a possible link to an increase in autoimmune diseases. Clin. Immunol..

[70] Zhang S., Liu Y., Wang X., Yang L., Li H., Wang Y., Liu M., Zhao X., Xie Y., Yang Y. (2020). SARS-CoV-2 binds platelet ACE2 to enhance thrombosis in COVID-19. J. Hematol. Oncol..

[71] Kaur S., Bansal R., Kollimuttathuillam S., Gowda A.M., Singh B., Mehta D., Maroules M. (2021). The looming storm: Blood and cytokines in COVID-19. Blood Rev..

[72] Greinacher A., Selleng K., Mayerle J., Palankar R., Wesche J., Reiche S., Aebischer A., Warkentin T.E., Muenchhoff M., Hellmuth J.C. (2021). Anti-SARS-CoV-2 spike protein and anti-platelet factor 4 antibody responses induced by COVID-19 disease and ChAdOx1 nCov-19 vaccination. Res. Sq..

[73] Corbett K.S., Edwards D.K., Leist S.R., Abiona O.M., Boyoglu-Barnum S., Gillespie R.A., Himansu S., Schäfer A., Ziwawo C.T., DiPiazza A.T. (2020). SARS-CoV-2 mRNA vaccine design enabled by prototype pathogen preparedness. Nature.

[74] Bos R., Rutten L., van der Lubbe J.E.M., Bakkers M.J.G., Hardenberg G., Wegmann F., Zuijdgeest D., de Wilde A.H., Koornneef A., Verwilligen A. (2020). Ad26 vector-based COVID-19 vaccine encoding a prefusion-stabilized SARS-CoV-2 Spike immunogen induces potent humoral and cellular immune responses. NPJ Vaccines.

[75] Vogel A.B., Kanevsky I., Che Y., Swanson K.A., Muik A., Vormehr M., Kranz L.M., Walzer K.C., Hein S., Güler A. (2020). A prefusion SARS-CoV-2 spike RNA vaccine is highly immunogenic and prevents lung infection in non-human primates. bioRxiv.

[76] Watanabe Y., Mendonça L., Allen E.R., Howe A., Lee M., Allen J.D., Chawla H., Pulido D., Donnellan F., Davies H. (2021). Native-like SARS-CoV-2 Spike Glycoprotein Expressed by ChAdOx1 nCoV-19/AZD1222 Vaccine. ACS Cent. Sci..

[77] Chen H., Xiang Z.Q., Li Y., Kurupati R.K., Jia B., Bian A., Zhou D.M., Hutnick N., Yuan S., Gray C. (2010). Adenovirus-based vaccines: Comparison of vectors from three species of adenoviridae. J. Virol..

[78] Mast T.C., Kierstead L., Gupta S.B., Nikas A.A., Kallas E.G., Novitsky V., Mbewe B., Pitisuttithum P., Schechter M., Vardas E. (2010). International epidemiology of human pre-existing adenovirus (Ad) type-5, type-6, type-26 and type-36 neutralizing antibodies: Correlates of high Ad5 titers and implications for potential HIV vaccine trials. Vaccine.

[79] Abbink P., Lemckert A.A.C., Ewald B.A., Lynch D.M., Denholtz M., Smits S., Holterman L., Damen I., Vogels R., Thorner A.R. (2007). Comparative seroprevalence and immunogenicity of six rare serotype recombinant adenovirus vaccine vectors from subgroups B and D. J. Virol..

[80] Nwanegbo E., Vardas E., Gao W., Whittle H., Sun H., Rowe D., Robbins P.D., Gambotto A. (2004). Prevalence of neutralizing antibodies to adenoviral serotypes 5 and 35 in the adult populations of The Gambia, South Africa, and the United States. Clin. Diagn. Lab. Immunol..

[81] Fausther-Bovendo H., Kobinger G.P. (2014). Pre-existing immunity against Ad vectors: Humoral, cellular, and innate response, what’s important?. Hum. Vaccin. Immunother..

[82] Barrett J.R., Belij-Rammerstorfer S., Dold C., Ewer K.J., Folegatti P.M., Gilbride C., Halkerston R., Hill J., Jenkin D., Stockdale L. (2021). Phase 1/2 trial of SARS-CoV-2 vaccine ChAdOx1 nCoV-19 with a booster dose induces multifunctional antibody responses. Nat. Med..

[83] Vinayagam S., Sattu K. (2020). SARS-CoV-2 and coagulation disorders in different organs. Life Sci..

[84] Martinez-Ribaya B., Areal F.J. (2020). Is there an opportunity for product differentiation between GM and non-GM soya-based products in Argentina?. Food Control.

[85] Bestle D., Heindl M.R., Limburg H., Van Lam van T., Pilgram O., Moulton H., Stein D.A., Hardes K., Eickmann M., Dolnik O. (2020). TMPRSS2 and furin are both essential for proteolytic activation of SARS-CoV-2 in human airway cells. Life Sci. Alliance.

[86] Xu S., Yang K., Li R., Zhang L. (2020). mRNA Vaccine Era-Mechanisms, Drug Platform and Clinical Prospection. Int. J. Mol. Sci..

[87] Pryzdial E.L.G., Lin B.H., Sutherland M.R., Gresele P., Kleiman N.S., Lopez J.A., Page C.P. (2017). Virus–Platelet Associations. Platelets in Thrombotic and Non-Thrombotic Disorders: Pathophysiology, Pharmacology and Therapeutics: An Update.

[88] Schubert S., Weyrich A.S., Rowley J.W. (2014). A tour through the transcriptional landscape of platelets. Blood.

[89] Salunkhe V., Papadopoulos P., Gutierrez L. (2015). Culture of Megakaryocytes from Human Peripheral Blood Mononuclear Cells. Bio-Protocol.

[90] Medicines & Healthcare Products Regulatory Agency. Summary of the Public Assessment Report for Pfizer/BioNTech COVID-19 Vaccine. https://www.gov.uk/government/publications/regulatory-approval-of-pfizer-biontech-vaccine-for-covid-19/summary-public-assessment-report-for-pfizerbiontech-covid-19-vaccine.

[91] Chapman L.M., Aggrey A.A., Field D.J., Srivastava K., Ture S., Yui K., Topham D.J., Baldwin W.M., Morrell C.N. (2012). Platelets present antigen in the context of MHC class I. J. Immunol..

[92] Almuqrin A., Davidson A.D., Williamson M.K., Lewis P.A., Heesom K.J., Morris S., Gilbert S.C., Matthews D.A. (2021). SARS-CoV-2 Vaccine ChAdOx1 nCoV-19 Infection Of Human Cell Lines Reveals Low Levels of Viral Backbone Gene Transcription Alongside Very High Levels of SARS-CoV-2 S Glycoprotein Gene Transcription. Genome Med..

